# Mechano-chemical and biological energetics of immobilized enzymes onto functionalized polymers and their applications

**DOI:** 10.1080/21655979.2022.2062526

**Published:** 2022-04-21

**Authors:** Tanvi Sharma, Changlei Xia, Abhishek Sharma, Pankaj Raizada, Pardeep Singh, Swati Sharma, Pooja Sharma, Sunil Kumar, SuShiung Lam, Ashok Kumar Nadda

**Affiliations:** a Department of Biotechnology and Bioinformatics, Jaypee University of Information Technology, Solan, India; b Center of Efficient Processing and Utilization of Forestry Resources, College of Materials Science and Engineering, Nanjing Forestry UniversityCo-Innovation, Nanjing,Jiangsu, China; c Department of Biotechnology, Himachal Pradesh University, Shimla, India; d School of Advanced Chemical Sciences, Shoolini University, Solan, India; e University Institute of Biotechnology, Chandigarh University, Gharuan Mohali, India; f CSIR-National Environmental Engineering Research Institute (CSIR-NEERI), Nagpur, India; g Higher Institution Centre of Excellence (Hicoe), Institute of Tropical Aquaculture and Fisheries (Akuatrop), Universiti Malaysia Terengganu, Kuala Nerus, Malaysia

**Keywords:** Enzyme, immobilization, functionalized polymers, applications

## Abstract

Enzymes of commercial importance, such as lipase, amylase, laccase, phytase, carbonic anhydrase, pectinase, maltase, glucose oxidase *etc*., show multifunctional features and have been extensively used in several fields including fine chemicals, environmental, pharmaceutical, cosmetics, energy, food industry, agriculture and nutraceutical *etc*. The deployment of biocatalyst in harsh industrial conditions has some limitations, such as poor stability. These drawbacks can be overcome by immobilizing the enzyme in order to boost the operational stability, catalytic activity along with facilitating the reuse of biocatalyst. Nowadays, functionalized polymers and composites have gained increasing attention as an innovative material for immobilizing the industrially important enzyme. The different types of polymeric materials and composites are pectin, agarose, cellulose, nanofibers, gelatin, and chitosan. The functionalization of these materials enhances the loading capacity of the enzyme by providing more functional groups to the polymeric material and hence enhancing the enzyme immobilization efficiency. However, appropriate coordination among the functionalized polymeric materials and enzymes of interest plays an important role in producing emerging biocatalysts with improved properties. The optimal coordination at a biological, physical, and chemical level is requisite to develop an industrial biocatalyst. Bio-catalysis has become vital aspect in pharmaceutical and chemical industries for synthesis of value-added chemicals. The present review describes the current advances in enzyme immobilization on functionalized polymers and composites. Furthermore, the applications of immobilized enzymes in various sectors including bioremediation, biosensor and biodiesel are also discussed.

## Graphical abstract

**Figure:**
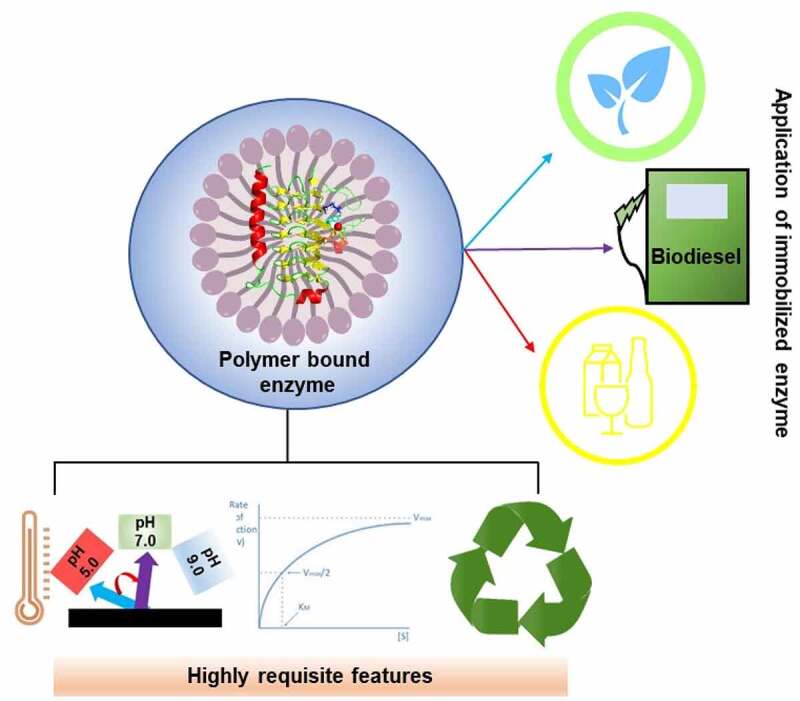

## Introduction

**Figure 1.**
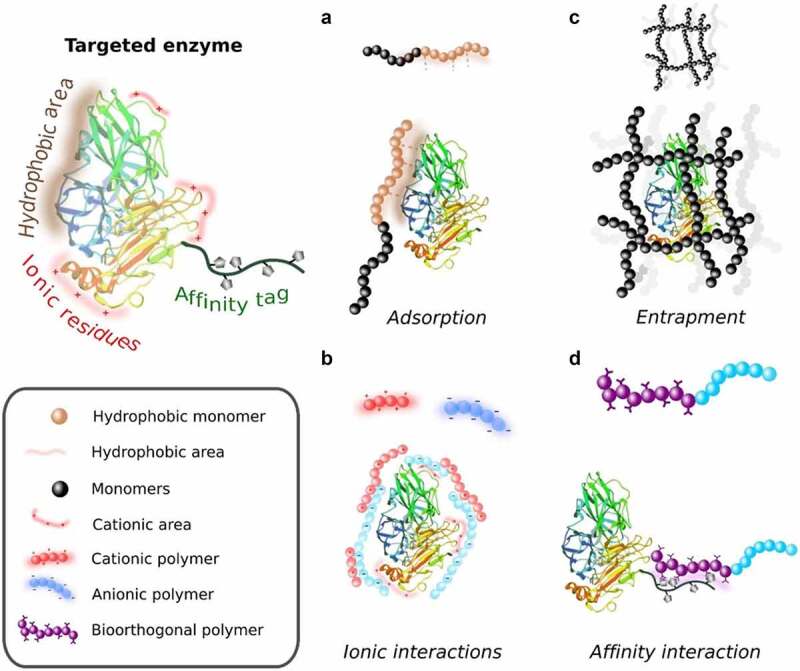
Schematic illustration of various types of immobilizations method. Reproduced with permission from rogriguez-abetxuko et al. 2020., frontier bioengineering biotechnology, 8, 830 [17].

Enzymatic transformations have appeared as a sustainable solution to create valuable products having plenty of applications in the industries including food, cosmetics, biomedicine, agrochemical, biochemical, biosensors, biofuels, *etc*. [1–5]. Notably, enzymes are ‘natural bio-catalyst’ having high chemo-selectivity, regioselectivity, and stereoselectivity. However, the use of enzymes in their native form possesses obstacles like low catalytic efficacy in no physiological reactions, high cost, instability, and difficulty in reuse [6]. Enzyme immobilization on solid materials can overcome the aforementioned drawbacks. In biotechnology, ‘enzyme immobilization’ is one of the methods by which biocatalyst attaches to the insoluble material. Furthermore, the rationale of immobilizing the enzyme is not only to re-use it but also to improve the enzyme properties, including stability, activity and turnover number, *etc* [6–9]. Moreover, the immobilized enzyme has improved stability and activity under harsh conditions like extreme pH and temperature as well as in the presence of organic solvents or substrates with enzyme inactivating features [10–12]. Furthermore, the application of immobilized enzymes in sustainable bioconversion of CO_2_, food waste and industrial by-products into value-added products may helpful to meet the objectives of circular bio-economy [13,14]. Although several enzyme immobilization methods do not employ any preexisting solid supports (crosslinked enzyme aggregates, crosslinked enzyme crystals, nanoflowers, crystals coated of enzymes, copolymers, sol-gels, enzyme trapping, *etc*.) [15]. The use of preexisting solids has some advantages so it is possible to select the mechanical properties of the biocatalyst independently of the enzyme modification, stabilization via multipoint covalent attachment may be quite effective, *etc*. In these protocols, the enzyme and matrix are two components that interact with each other by various binding forces, such as cross-linking, entrapment, absorption, encapsulation, and covalent bonding (Figure 1) [10,16].

**Figure 2.**
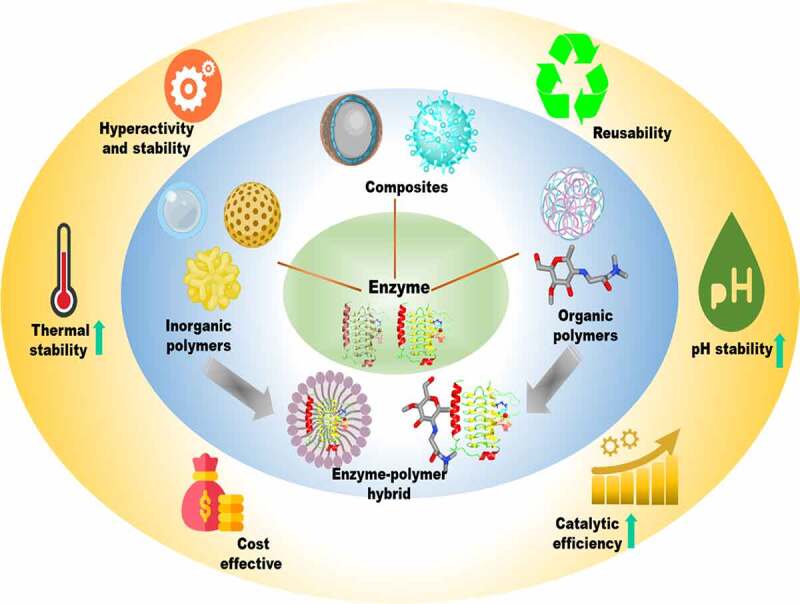
Immobilization of enzymes onto functionalized polymeric matrices and the advantages in terms of improving the physicochemical properties of immobilized enzymes.

In general, the immobilization matrix can be classified into organic or inorganic types, and the organic matrices are further classified into synthetic polymers (polystyrene, polyacrylamides, or polyamides) and biopolymers (dextran, chitosan, agarose, pectin, gelatin) (Figure 2) [17–20]. Previously, de Souza and his coworkers immobilized lipase on modified cellulose for ester synthesis. The immobilized enzyme was used to esterify oleic acid and ethanol and it shows 97% conversion at 60°C [21]. Likewise, Moreira et al., evaluated the lipase immobilized on magnetic nanoparticles for production of ester and the immobilized lipase showed 81.7% conversion at 40°C [22,23]. The lipase immobilized on polymeric matrix *i.e*., cellulose showed a higher conversion rate at higher temperature as compared to lipase immobilized on nanoparticles. Moreover, the important factors considered during the immobilization of enzymes are purity, functional moieties on its surface, and molecular mass [24]. Also, the functional moiety on the biocatalyst surface gives an idea about which type of interaction takes place between the enzyme and matrix. Furthermore, the efficiency of polymeric matrices can be enhanced by surface functionalization [25]. The surface modification allows the addition of a particular functional group onto the surface of the polymeric matrix to attain the desired properties. Most of the matrices used for enzyme immobilization are hydrophilic and which could lead to weak interaction with biocatalyst, thus the polymeric matrices need to be functionalized. The polymeric matrices can be functionalized with various functional groups including diethylaminoethyl, carboxymethyl, and quaternary ammonium derivatives [26,27]. Also, the surface modification can notably alter the catalytic performance of the immobilized biocatalyst by altering their dispersion capability and interaction with the biocatalyst. Mostly, surface functionalization is used to impart stability and biocompatibility to the polymeric matrix for application purposes [28–31]. Hence, enzyme immobilization has become almost compulsory for designing an enzyme as an industrial biocatalyst. For industrial application, the cost of immobilized enzyme depends on reaction kinetics, specificity and number of times the enzyme is reused. The guidelines that are provided to evaluate the enzyme production cost for commodity products lies between 2000 and 10,000 kg product/kg of immobilized enzyme, and for pharma products 50–100 kg product/ kg immobilized enzyme. Before scaling up the processes using immobilized biocatalyst, the economic estimation of the process should be performed, considering the cost of each factors including native enzyme, reactors, matrix for enzyme immobilization, and downstream processing [32]. In this review article, we comprehensively discussed the use of polymeric matrices for enzyme immobilization. For better understanding, we first described the pros and cons of polymeric matrices and then illustrated the energetics kinetics of the polymer-bound enzymes. At last, their potential applications of polymer bound enzymes in biocatalysis and enzyme technology have been emphasized.

## 2. Pros and cons of matrices as immobilization platforms

The selection of suitable matrices for immobilizing the enzyme can affect the practicality of its industrial applications. For immobilization, matrices must fulfill the prerequisite of biocatalyst to be immobilized and able to withstand the enzymatic reaction conditions [33,34]. In particular, biopolymeric matrices become the apparent choice of support due to their unique features such as biodegradable, nontoxic, economical, and outstanding binding with an enzyme [35,36]. The pros and cons of polymeric immobilization matrices are listed in Table 1. Various polymeric matrices having functional entities have been utilized for the immobilization of enzymes. For example, in previous studies lipase was immobilized on cellulosic polyurethane via hydroxyl group that enhanced its mechanical and surface properties [37,38]. Moreover, a synthetic polymer can be used as potent matrices because the presence of the polymer layer shields the active center of the biocatalyst from adverse effects of reaction conditions. Recently, Ma and coworkers [39] co-immobilized the hemins and glucose oxidase in poly(vinyl alcohol) (PVA) aerogel and their findings suggest that immobilized enzyme resists the denaturation at a higher temperature. Previously, the lipase was immobilized on nonporous polystyrene and the results showed that immobilized lipase exhibited better thermal stability, as immobilized lipase was less sensitive to thermal deactivation due to conformational changes in its structure [40]. However, the use of synthetic polymer has limitations such as high production cost, time-consuming, and complicated synthesis process. Previously, Kim and his coworker developed biocompatible cellulose nano-crystal prepared from bacterial cellulose and lingo-cellulose. And used these cellulose nano-crystals for lipase immobilization, the resulting biocatalytic system exhibited higher immobilization yield and improved thermal stability [41,42]. Traditionally, inorganic polymers such as mesoporous silica have a large surface area (>700 m^2^/g) for enzyme immobilization [43]. Also, in recent studies, functionalized activated carbon with high surface area and porous structure was used for lipase immobilization and the immobilized biocatalyst exhibited higher operational stability than free enzyme [44,45]. Furthermore, Mohammadi et al. [46] immobilized a laccase on to functionalized silica *via* covalent binding, and their study showed that immobilized laccase was highly stable and exhibited good removal efficiency for phenolic compounds. The aforementioned studies support that inorganic polymer are categorized by good sorption properties due to their porous nature and large surface area that offer the various binding sites for enzyme immobilization. These polymers are also well-known for excellent mechanical durability as well as higher chemical and thermal stability. Nevertheless, there are limitations of using inorganic polymeric matrices such as limited biocompatibility and cross-linking agents are required to form covalent bond weak matrices and enzymes [47,48]. Moreover, the protein structure of enzymes immobilized on some of polymeric matrix got distorted, when exposed to hydrophobic surface or organic solvents and gas bubble. To protect the enzyme from this negative effect, the coating of immobilized enzyme by hydrophilic polymers may be an alternative. Furthermore, enzyme immobilization using multipoint covalent attachment on preexisting matrix is an effective method to produce more rigid enzyme polymer for industrial applications [49,50]. Nowadays, researchers are interested to blend organic and inorganic matrices as this type of composite possesses the advantageous features of both matrices. These composite supports material shows exceptional stability and protects the enzyme’s three-dimensional structure during immobilization. For example, blending biopolymer (having a high affinity to an enzyme) with a synthetic polymer (known for its mechanical stability or thermal resistance) and using this composite as biocatalyst matrices resulted in reusable and stable enzymatic system [51]. Hence, the resultant biocatalytic system exhibits the features not detected in their counterparts and can be utilized in various applications, such as environmental remediation, pharmaceutical, and food industry.

**Table 1.** Advantages/disadvantages of polymeric matrices

S.No	Polymeric matrices	Advantages	Disadvantages
1.	**Synthetic polymer**	Presence of several functional groups Strong binding of an enzyme Immobilization of greater amount of enzyme	Time-consuming Nonrenewable High production cost
2.	**Biopolymer**	Nontoxic Cheap High affinity for enzymes Biodegradable Presence of several functional groups Reuseable	The necessity of clearing and proper preparation
3.	**Inorganic polymers**	High stability Mechanical resistance Good sorption capacity Easy surface functionalization Relatively cheap Inert	Possibility of unspecific interaction Limited biocompatibility Weak enzyme-matrices interaction without crosslinking agents
4.	**Composites**	Strong affinity for the enzyme Highly stable Limited enzyme leakage	Expensive Limited reusability

## 3. Effects of functionalization of polymeric matrices with various organic and inorganic groups

**Figure 3.**
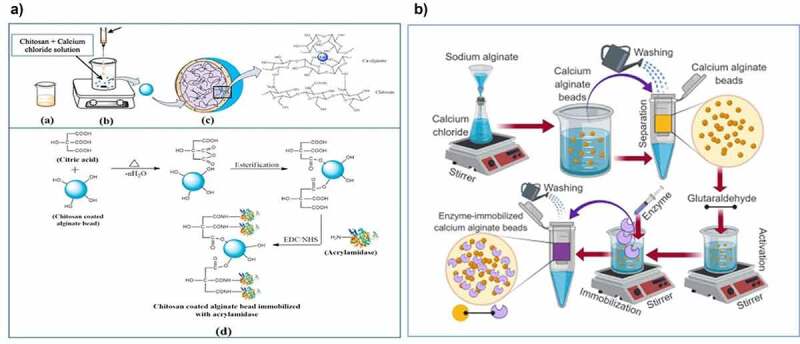
The overall scheme of A) immobilization of acrylamidase on chitosan. Reproduced with permission from Bedade et al. 2019., food chemistry, 275, 95–104 .[58]. B) immobilization of lignin peroxidase on sodium alginate beads. Reproduced with permission from Bilal et al. 2019, biocatalysis and agricultural biotechnology, 20,101,205 [59].

The inert nature of polymeric material limits its application in several industrial realms. Furthermore, the functionalization of the polymeric matrix involves the grafting of specific functional moieties onto its surface to obtain a polymeric matrix with desired properties. The functionalization results in tuning the surface properties including hydrophilicity, protection from chemicals, and modification of the surface reactivity [52,53]. Functionalization can affect their interaction with biocatalysts, thus it may result in changing the activity of immobilized catalysts [53]. There was an interesting study describing the immobilization of pectinase onto hydrogel composed of alginate and agar, then activated by glutaraldehyde to incorporate the aldehyde group. Another study revealed that immobilized pectinase had improved reusability and the optimum temperature was also shifted from 55 to 60°C [54]. The covalent bond formation between the matrix and enzyme results in increasing the rigidity of the 3D structure of an enzyme, which in turn shifting its temperature optima too. Furthermore, amylase was immobilized onto magnetic chitosan modified with glyoxal, epichlorohydrin, and glutaraldehyde to avoid enzyme leakage and improve its mechanical stability. The excellent thermal, storage stability, immobilization efficiency, and reusability were observed for immobilized enzymes. This is because the aldehyde (-CHO) group in glyoxal/glutaraldehyde activated chitosan bound with functional moieties of amino acids (-NH_2_), whereas epoxy moieties in epichlorohydrin activated chitosan bound with -OH, -NH functional moieties on the biocatalyst [55]. Previously, a lipase was covalently immobilized on glutaraldehyde-activated chitosan, and the resultant biocatalyst showed a high immobilization yield but a decrease in the catalytic activity. Glutaraldehyde may immobilize lipase using three different mechanisms including interfacial activation, covalent attachment, and ion exchange, so a huge amount of biocatalyst binds to the support and leads to enhanced immobilization yield. The binding of chitosan to the lipase may distort its 3-D structure resulting in a decrease in enzyme activity [56]. Later there was research describing immobilization of cellulase onto APTES functionalized silica gel cross-linked using glutaraldehyde. The immobilized enzyme exhibited high reusability after modification with APTES, because the active amino moieties were formed that results in covalent binding of an enzyme with support, thus preventing the leaching of cellulase [57]. Moreover, in a previous study reported by Bedade et al. [58] for the removal of acrylamide from coffee acrylamidase was immobilized on to the chitosan-coated alginate beads activated by citric acid. The immobilized acrylamidase exhibited improved thermal stability and retained 80% of activity after four cycles of reuse (Figure 3a). The improved thermal stability may be referred to as the covalent attachment of citric acid with acrylamidase, thus resulting in increased rigidity of the enzyme.

Moreover, lignin peroxidase had been covalently immobilized on glutaraldehyde-activated alginate beads and then efficiently utilized for decolorization of textile dye, which suggests its utility in bioremediation purposes [59] (Figure 3b). Alatawi and coworkers [60], immobilized urease on amino-functionalized cross-linked carboxymethylcellulose. The thermal stability was enhanced after immobilization due to the formation of the covalent bond between urease and support, which can prevent the conformational changes upon the further rise in temperature. Glutaraldehyde is a mostly used cross-linking reagent, but it is toxic and harmful to living cells so that limit its application in the food and dairy industry [61]. To develop a safer bioprocess for the dairy industry, β-galactosidase was immobilized on chitosan functionalized using genipin, a natural cross-linker agent [62]. Remarkable outcomes of catalytic activity were attained at pH 4.0 and 60°C with enhanced stability and retaining 100% of activity after 25 repetitions cycles of lactose hydrolysis. Furthermore, a molecularly imprinted polymer (MIP) was synthesized by utilizing boric acid-modified iron nano-particles by silane emulsion self-assembly method for immobilization of horseradish peroxidase. The enzyme was covalently bound with boronic acid groups and successfully utilized to develop the visual sensors for sarcosine and glucose detection [63]. These studies suggested that functionalized polymeric matrices provide more functional groups for enzyme molecules to bind, thus immobilized enzymes showed higher activity and stability under harsh reaction conditions. During functionalization of the matrix, the main challenge is to create a strong attachment of biocatalysts by retaining their functionality and catalytic activity.

## 4. Energetics and kinetic behavior of polymer immobilized enzymes

**Figure 4.**
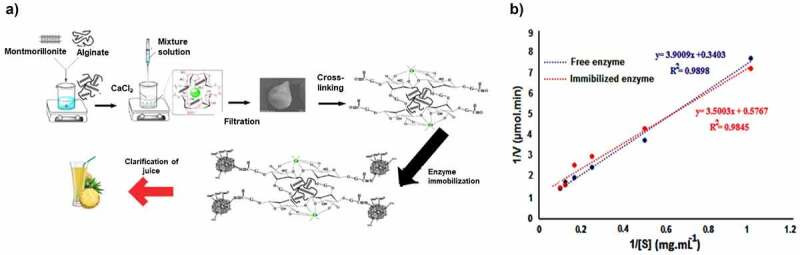
Schematic illustration for the immobilization of pectinase on alginate-montmorillonite (MMT) beads b) lineweaver burk plot of free and immobilized pectinase. Reproduced with permission from Mohammadi et al. 2019, international journal of biological macromolecules, 137, 253–260 [71].

To uplift the laboratory scale research to the industrial-scale application with enhanced productivity, it is essential to study and consider the kinetic parameters of an enzyme. The enzyme kinetics provides information about enzyme reaction rates, enzyme catalytic mechanism, and how an enzyme inhibits a drug [64]. For example, the Michaelis-Menten equation is regularly used to determine the effect of immobilization on enzyme activity. The kinetic parameters of immobilized biocatalyst including turnover number (*K*_cat_), maximum velocity (*V*_max_), and Michaelis Menten constant (*K*_m_), can vary from that of the free biocatalyst [65,66]. However, the immobilization method may affect the kinetic behavior of biocatalyst due to various factors such as limited access to the active center, enzyme conformational changes, and deviation in the microenvironment around the immobilized enzyme *etc* [67]. The changes in kinetic parameters such as *K*_m_ and *V*_max_ helps to determine the success of the immobilization process. In a recent study, Alnadari et al. [68] found that the *V*_max_ and *K*_m_ value of free biocatalyst was 31.0 Umg^−^[1] and 2.7 mM, but the *V*_max_ value decreased to 26.6 Umg^−^[1], and *K*_m_ value increased to 2.7 mM after the immobilization of β-glucosidase on to chitin functionalized nanoparticles with *p-*NPG as substrate (Table 2)., The immobilization process ameliorates the hindrance of biocatalyst toward its substrate and thus moderately increases the *K*_m_ value. Furthermore, pectin methylesterase, pectin lyase (PL), and polygalacturonase (PG) were immobilized on the chitosan cross-linked using dextran polyaldehyde. The results showed a slight difference between the *V*_max_ and *K*_m_ value of free and immobilized biocatalyst which might be due to immobilized enzyme protecting its 3D structure after immobilization [69]. Aslam et al. [70] obtained *V*_max_ of 308 Uml^−^[1] and 597.0 Uml^−^[1] accompanied by *K*_m_ values of 111.0 and 120.0 μM for free and chitosan immobilized laccase, respectively. After immobilization, a slight increase in *V*_max_ corroborated the increase in catalytic efficiency of the immobilized enzyme. In a previous study, pectinase was covalently immobilized on alginate-montmorillonite beads and exhibited a decline in *K*_m_ value, indicating that the enzyme affinity to the substrate has been enhanced (Figure 4) [71].

**Table 2.** Kinetic parameters for the enzymatic reactions

S.No	Enzyme	Polymeric matrices	*K*m (mM)	*K*_cat_ (s^−1^)	*K*_cat_/*K*_m_ (mM^−1^s^−^[1])	*V*_max_	Reference
1	Laccase	Chitosan	0.33	55.8	169.2	0.14^a^	[75]
2	Acid phosphatase	Gelatin	0.3	-	-	2.1 ^b^	[76]
3	β-glucosidase	Chitin funcmethanoltionalized nanoparticles	3.31	25.8	7.8	-	[68]
4	Formate dehyrogenase	Polyethylenimine grafted graphene oxide	6.07	3.2	0.5	0.0123^a^	[77]
5.	Urease	Carboxymethyl cellulose	14.0	-	-	2.0^b^	[60]
6	Laccase	Chitosan	0.12	-	-	597.0^c^	[70]
7	Acid phosphatase	Agarose	0.23	-	-	2.3 ^b^	[78]
8	Carbonic anhydrase	Polyurethane	12.2	2.0	166.4	-	[79]
9	Lipase	Polymer-grafted silica nanoparticles	2.04	296.0	145.0	15.1^d^	[80]
10	Lipase	Magnetic cellulose nanocrystal	12.4	-	-	0.12 ^d^	[81]
11	Carbonic anhydrase	Mesoporous aluminosilicates	0.15	1.9	-	2.3 ^e^	[82]
12	Invertase	Chitosan	61.3	-	-	177.7 ^b^	[83]
13	β-galactosidases	Collagen	6.9	8456.0	1224.0	8670.0^d^	[76]
14	Maltase	Agarose	1.91	-	-	6214.0^f^	[84]
15	Lactase	Agarose-carboxymethyl cellulose	107.24	36.6	-	-	[74]
16	Laccase	Chitosan-clay composite	0.5	-	-	96.0 ^f^	[85]
17	Tyrosinase	Polyamide	1.56	-	–	-	[86]
18	Lipase	Agarose	261.7	1,806,241.8	6900.0	46.3^g^	[87]

^a^µMmin^−1^ ml^−1^

^b^µmol^−1^min^−1^mg

^c^UmL^−^[1]

^d^mMs^−^[1]

^e^molmin^−1^ml^−1^

^f^U ml^−1^ min^−1^

^g^IUml^−^[1]

Polygalacturonase and pectin lyase (PL) were immobilized on the chitosan and results showed a very slight difference between *K*_m_ and *V*_max_ in immobilized as well as free form indicating that there was no conformational change in enzyme after immobilization [72]. Furthermore, Jaswal and his coworkers successfully entrapped pectinase in polyvinyl alcohol and found that *K*_m_ and *V*_max_ of immobilized enzyme were changed significantly [73]. Lactase was immobilized in carboxyl methyl cellulose-alginate gel exhibiting increased *K*_m_ of 107.24 mM, while the *K*_cat_/*K*_m_ and *K*_cat_ of immobilized biocatalysts were similar to that of free enzyme [74]. The increased *K*_m_ indicates the lower affinity of the immobilized biocatalyst for a substrate that may be because of low accessibility of substrate to the active center, loss of biocatalyst flexibility, and diffusion resistance to substrate transport.

Bindu et al. [55] studied thermodynamics and kinetic properties of amylase immobilized on magnetic chitosan and found that immobilized amylase had higher enthalpy and free energy as compared to free amylase. The thermodynamic studies can give information about the heat of the inactivation of immobilized and free enzymes. Whereas noteworthy enhancement in the *K*_m_ (0.65 mg/ml) was observed as compared to the native enzyme (0.45 mg/ml), indicating the efficacy of immobilization methods. Previously, *V*_max_ and *K*_m_ values were calculated for *β*-glucosidase entrapped in the alginate using the Lineweaver-Burk plot. For the immobilized enzyme, a decrease in *V*_max_ (0.745 *μ*mol min^−1^ ml^−1^) was observed as compared to the free counterpart (0.94 *μ*mol min^−1^ ml^−1^). This decline might be attributed to the interaction of biocatalyst with functional moieties present on the surface of beads or limited access of substrate to the active center of the enzyme. However, the increase in *K*_m_ value after immobilization was observed and this may be caused by diffusional resistance, steric hindrance, and loss of enzyme flexibility [88]. Catalase was immobilized onto chitosan beads and showed that *V*_max_ of free catalase (33,000 μmol min ^−^[1] mg^−^[1]) was higher than immobilized catalase (26,300 μmol min ^−^[1] mg^−^[1]), due to conformational alterations in enzyme during immobilization [89]. To overcome the drawbacks of chitosan beads such as low density, Mardani et al. [90] utilized the chitosan-montmorrillonite beads for amylase immobilization. The results showed that the *K*_m_ value of immobilization was higher as compared to free enzyme. The research literature regarding the kinetic analysis showed that in most of the studies enzyme affinity toward substrate *i.e., K*_m_ usually increases, whereas *V*_max_ decreases after immobilization that might be due to conformational changes in a enzyme structure.

## 5. Polymeric matrices immobilized enzymes for industrial bio-catalysis

Recently, polymers attained from renewable resources such as agarose, alginate, chitosan, and cellulose from marine algae, brown algae, crustacean skeleton, bacteria, and plants have engrossed much attention owing to their abundance and interesting properties such as biodegradability, nontoxicity, flexibility, and availability of several active sites for incorporating new functionalities [91,92]. To date, renewable polymeric matrices, like gelatin, starch, alginate, pectin, cellulose, and chitosan are commonly used for enzyme immobilization [36,93–95]. Furthermore, a large number of biopolymers shows potential as an ideal matrix for various applications in the biofuel, environmental, food, biomedical, and pharmaceutical sectors.

### 5.1. Biodiesel and bio-energy generation

Biodiesel is considered a renewable transportation fuel due to its excellent properties such as biodegradability, nontoxicity, and bio-renewability. Biodiesel can be directly utilized in a compression ignition engine without modification [96,97]. Biodiesel production using a chemical catalyst is an energy-intensive process and also generates wastewater. The use of lipolytic enzymes for biodiesel production is economically and environmentally more beneficial than using chemical catalysts [23,98,99]. Microorganisms-derived lipase is utilized for biodiesel production including *Candida rugosa, Aspergillus Niger, Pseudomonas cepacian, Rhizopus oryzae, Burkholderia cepacia, Pseudomonas cepacia, Rhizopus miehei, and Thermomyces lanuginosus* [100]. The major disadvantages of using free lipase for biodiesel production are high cost and low yield. Typically, biodiesel is synthesized in low water content, so immobilization avoids agglomeration of the enzyme. The *Pseudomonas cepacian* lipase was immobilized on bio-support beads (polyvinyl alcohol and alginate) and utilized for transesterification of castor and Karanja oil. The reusability assay of immobilized biocatalyst showed a 52% loss in the biodiesel yield after nine cycles of repeated use and this might be due to the leaching of lipase from alginate beads [101]. Furthermore, various researchers have reported the conversion of food waste into biodiesel using an immobilized enzyme. For *e.g*., Khan et al. [102] immobilized lipase on chitosan beads applied for biodiesel synthesis from waste cooking oil. Along with good catalytic efficiency, the resultant biocatalyst showed more than 90% biodiesel yield. The transformation of food waste into biodiesel not only offers economic benefits but also mitigates the food waste decomposition problems. Furthermore, lipase covalently immobilized on to poly-porous magnetic cellulose showed 85% of biodiesel yield up to the fourth cycle, and after that its yield declined. The possible reason for the decline in catalytic activity could be mass transfer limitation, structural changes in lipase active center, and nonspecific attachment [103]. Recently, Muanruksa and his coworkers [104] entrapped lipases onto pectin-alginate (PA) and gelatin alginate (GA) hydrogel to produce biodiesel from waste frying oil. Both the immobilized biocatalysts showed biodiesel yield between 75 and 78.3%, and the PA immobilized lipase had higher residual activity after seven cycles of reuse as compared to GA immobilized lipase, which may be due to gelatin-alginate network degradation after reuse. And the entrapped lipase in PA might show more conformational flexibility and it was not degraded by physical and chemical forces. In a previous study, Romdhane et al. [105] used chitosan immobilized lipase for biodiesel synthesis from waste cooking oil. In a fixed bed reactor biodiesel yield reached 92% in 24 h batch reaction. The high biodiesel yield might be due to the polymeric backbone chain of chitosan providing structural support to covalently linked lipase. Moreover, in a covalent attachment, a strong chemical bond is formed that ensures negligible leakage of lipase from the support. Moreover, the main challenge for biodiesel enzymatic production is the cost of lipase, but immobilization allows the reuse of the enzyme and makes continuous production feasible. Till now, the commercial biodiesel production is in the preliminary stage, and more continuous reactors research is needed. Thus, enzymatic biodiesel production could be extended to more processing plants as an eco-friendly renewable energy source.

### 5.2. Food, dairy, and confectionery industry

**Figure 5.**
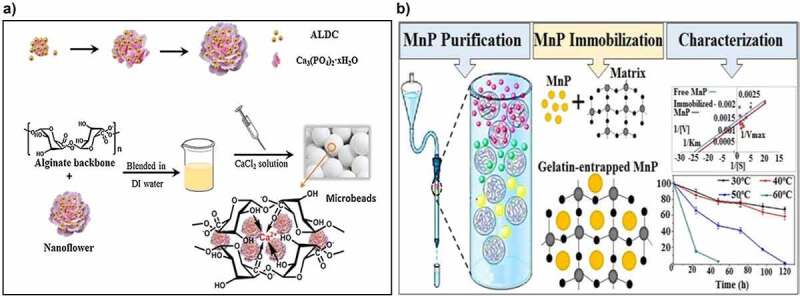
A) Schematic representation for the synthesis of enzyme inorganic nanoflower/alginate beads. reproduced with permission from Zhao et al. 2017., process biochemistry, 57, 87–94 [114]. B) immobilization of manganese peroxidase on glutaraldehyde-activated gelatin for fruit juice clarification. Reproduced with permission from Bilal et al. 2016., Catalysis letter, 146, 2221–2228 [123].

Immobilized enzymes are widely utilized in food processes such as winemaking, production of fructose syrups, and lactose hydrolysis [106,107](Table 3). In the food industry, biocatalysts are used to enhance the sensory properties including texture, flavor as well as to provide nutritional value to the products [107–110]. For example, pectinase is involved in the breakdown of pectin in fruit pulp, and it enhances the fruit juice yields and provides clarification of juice. Recently, Tasgin et al. [111] covalently immobilized pectinase in carboxymethyl cellulose and applied it for fruit juice clarification. Notably, the clarification percentage of immobilized pectinase reached 23% than that of free enzyme and exhibited good reusability. Inulinase from *Aspergillus tubingensis* was immobilized on chitosan particles for hydrolyzing inulin to enhance the production of fructose syrup. The immobilized enzyme exhibited 95% of inulin hydrolysis yield even at high concentrations (17.5%) [112,113]. The utilization of immobilized inulinase for hydrolysis can be a cost-effective approach for the large-scale production of fructose. Moreover, Zhao and his coworkers synthesized the nanoflower/alginate beads to immobilize acetolactate decarboxylase for preventing the diacetyl formation in beer fermentation (Figure 5a). It was found that the diacetyl concentration declined to 0.1 ppm because the immobilized enzyme converts acetolactate into flavorless acetoin [114].

**Table 3.** Polymeric matrices immobilized enzyme and biotechnological applications

S No	Enzyme	Source	Renewable matrix	Mode of immobili- -zation	*K*cat Free enzyme	*K*cat Immobilized enzyme	Enzymatic performance	Application	References
1.	β-galactosidase	*Cicer* *arietinum*	Agarose	Adsorption	3.13 × 10^−^[^4]^	4.15 ×10^−^[^4]^	High tolerance in acidic and alkaline pH and improved reusability	Lactose hydrolysis	[126]
2.	Pectinase	*Bacillus* *licheniformis*	Agar-agar	Entrapment	NA	NA	Retained 80% activity at 40°C and exhibited high reusability for pectin hydrolysis	Fruit juice industry	[127]
3.	Alcohol dehydrogenase	*Saccharomyces* *cerevisiae*	Gelatin	Encapsulation	NA	NA	Improved pH tolerance, recycling, and storage stability	Brewing industries	[128]
4.	Phytase	*Lactarius volumes*	Modified chitosan	Covalent binding	NA	NA	Enhanced catalytic activity and resistance to metal ions	Hydrolysis of phytic acid in cereals	[129]
5.	Lipase	*Candida rugosa*	Agarose	Covalent binding	NA	NA	Improved stability and retained recycling activity up to four cycles of the reuse	Hydrolysis of fat	[130]
6.	Pectinase	*Acinetobacter calcoaceticus*	Cellulose	Covalent binding	NA	NA	Enhanced efficiency and reusability	Fruit juice clarification	[111]
7.	β-glucosidase	*Bacillus subtilis*	Alginate	Entrapment			Improved reusability and storage stability	Sugarcane juice	[88]
8.	Inulinase	*Aspergillus tubingensis*	Chitosan	Covalent binding	NA	NA	Enhanced stability and more than 70% chicory inulin were hydrolyzed to fructose at 60°C	Production of fructose syrup	[112]
9.	Acrylamidase	*Cupriavidusoxalaticus*	Chitosan coated calcium alginate	Covalent binding	NA	NA	Better pH and thermal stability	Removal of acrylamide from roasted coffee	[58]
10.	Maltase	*B. licheniformis*	Agar-agar	Entrapment	NA	NA	Retained 100% of activity after 2 hr at 50°C	Maltose hydrolysis	[124]
11.	Amylase	*Bacillus amyloliquefaciens*	Alginate	Entrapment	NA	NA	Retained 60% of initial activity after 5 cycles of reuse	Starch hydrolysis	[122]
12.	Pectinase	*Bacillus licheniformis*	Chitosan	Covalent binding	NA	NA	Enhanced pH and thermal stability	Pectin polymer degradation	[131]
13.	Inulinase	*Aspergillus Niger*	Chitosan	Covalent binding	NA	NA	Improved pH and temperature tolerance	Inulin hydrolysis	[132]
14.	Glucose oxidase	*Aspergillus Niger*	Alginate	Encapsulation	NA	NA	Retained 92% of activity at pH 4.0 and reused up to 7 cycles	Reduction of fermentable sugar in the must	[133]
15.	Transglutaminase	*Serratia plymuthica*	Alginate	Cross-linking	NA	NA	Enhanced stability and isomaltulose yield	Sucrose conversion into isomaltulose	[134]
16.	Pectinase	*Aspergillus* *aculeatus*	Calcium alginate beads	Entrapment	16.45	6.07	Excellent operational stability retained 80% of activity up to three cycles of reuse	Fruit juice clarifiation	[135]
18.	Manganese peroxidase	*Ganoderma* *lucidum*	Gelatin	Encapsulation	NA	NA	Improved thermal stability and reusability	Fruit Juice Clarification	[123]

**Figure 6.**
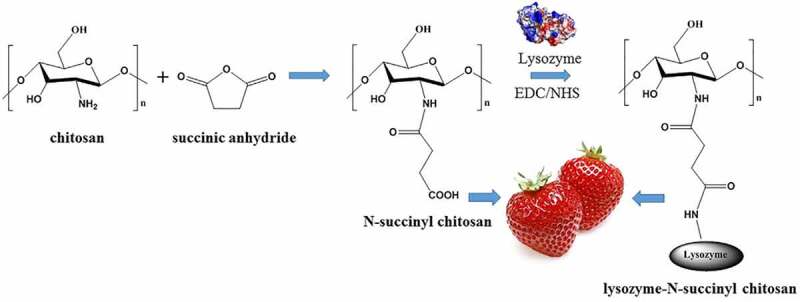
Illustration of N-succinyl chitosan preparation and its application strawberry preservation. Reproduced with permission from Niu et al. 2020., Food Control, 106, 829. [125].

Allulose is a ‘sweetener having zero-calorie’ and has a sweetness level is similar to dextrose. In 2014, Tate & Lyle developed the industrial process for the synthesis of the allulose using improved epimerase immobilized on ion exchange resin [115]. The economic benefits of using ion exchange resin for immobilizing the enzyme in food applications is the possibility of resins to regenerate using cost-effective reagents such as NaOH and HCl. In 2017, allulose was launched in the market named as DolciaPrima using a similar manufacturing process [116]. In the dairy industry, galactosidase catalyzes the hydrolysis of lactose and plays an important role in the manufacturing of lactose-free products [117]. For example, Klein et al. [118] immobilized a galactosidase in chitosan microparticles, and in the packed bed reactor the resultant biocatalyst hydrolyzed ~90% of lactose at 37°C. Galactosidase covalently immobilized on a functionalized agarose gel was applied for hydrolyzing the lactose. Notably, the immobilized biocatalyst showed higher lactose hydrolysis efficacy of 83%[119]. As the immobilized galactosidase exhibited acidic pH optima (4.5–5.0) that are suitable for whey processing, whereas the enzyme having neutral pH optima is good for milk processing. Furthermore, Snow Brand Milk Products in Japan reported the method for hydrolysis of lactose using *S. lactis* galactosidase entrapped in cellulose triacetate fibers. This plant was able to convert ten tons of milk/per day and cellulose immobilized galactosidase that showed less than 9% of activity even after 50 cycles of reuse [120,121]. Indeed, amylase entrapped in calcium alginate beads was applied for starch hydrolysis. The hydrolysis ability of entrapped amylase was found to be 10–20% less as compared to free amylase and this may be due to the gel matrix of calcium alginate beads interfering with the diffusion of a starch molecule to the active center of biocatalyst [122]. For instance, Bilal et al. [123] have proposed a cost-effective approach to immobilize manganese peroxidase on glutaraldehyde-activated gelatin for fruit juice clarification. Glutaraldehyde reacts with an amino group of protein and forms a spacer arm between matrix and biocatalyst by Michael type addition or by Schiff base formation. Experimental data analysis in previous reports showed that immobilized enzymes had improved thermal stability and catalytic activity (Figure 5b). Moreover, Gangadharan et al. [124] immobilized maltase in agar-agar for degradation of maltose, they found that entrapped maltase retained 50% of original activity up to 5 cycles of reuse and after that, it started decreasing due to the leaching of an enzyme. Previously, Niu and his coworkers immobilized lysozyme on N-succinyl chitosan, to obtain nontoxic and green strawberry preservative (Figure 6). The lysozyme breaks the β-1-4-glycosidic bond in the bacterial cell wall and has an antibacterial effect on gram-positive bacteria. They found that the storage life of strawberries was prolonged up to three days when treated with immobilized lysozyme. [125]. In the food industry, polymeric matrices are preferred as they are nontoxic and easily accessible. The polymeric immobilized enzyme offers advantages over free enzyme in terms of operational stability, large-scale applicability, and catalytic efficiency. The main drawback of using immobilized enzymes in the food industry is the microbial contamination of enzymes, so there is a need to propose a specific method to control this.

### 5.3. Environmental remediation

The release of environmentally worrisome compounds such as heavy metals, dyes, nitrates, carbon dioxide, antibiotics, heavy metals and naphthol can pose a hazard to the environment and human health. Many of these compounds are carcinogenic, mutagenic, and harmful that may cause problems for marine and human live [136]. For the remediation of effluents released from various industries usually requires a method *i.e*., cost-effective, efficient, and eco-friendly.

#### 5.3.1 Environmental waste treatment using polymeric matrics immobilized enzyme

**Figure 7.**
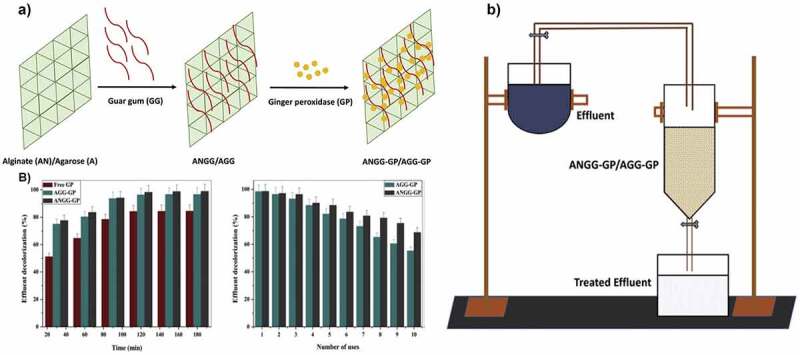
a) The scheme of ginger peroxidase (GP) immobilization in ANGG/AGG b) Effluent decolorization in stirred batch reactor and reusability of immobilized GP in ANGG/AGG c) Schematic representation of continuous reactor. Reproduced with permission from Ali et al. 2018., International Journal of Biological Macromolecules, 116, 463–471 [145].

Heavy metals, hazardous and pharmaceutical pollutants are released from industries into water bodies causing serious problems to human beings and the marine ecosystem [137,138]. The attention of environmental scientists has been attracted toward immobilized enzymes for removing anilines, dyes, heavy metals, and pharmaceutical pollutant [139–141]. In recent years, laccase, lignin peroxidase, lipase, and horseradish peroxidase are most commonly utilized for chemical pollutants degradation and usually catalyze the oxidation of various compounds as compared to the oxidative enzymes [110,142–144]. The enzyme, such as lignin peroxidase, has been covalently immobilized on glutaraldehyde-activated alginate beads and then efficiently utilized for the decolorization of textile dye. Notably, the dye decolorization efficiency of immobilized laccase was 80% even after the 5^th^ cycle [59]. After the 5^th^ cycle of reuse, the decrease in catalytic performance was observed which might be due to the interference of free radical in the enzyme active site, which leads to enzyme inhibition. Previously, Ali and Husain entrapped ginger peroxidase in alginate/agarose blended with gum for decolorization of textile effluent (Figure 7). The immobilized enzyme was effective in decolorizing 90% of color from the textile industry up to 10 days of operation which might be due to the hydrophilic nature of gum which increases the molecular dimension of an enzyme by forming a complex with it and retained inside the support for a long time [145]. Furthermore, Zhou and coworkers immobilized rhodanese on alginate beads and employed it for the biodegradation of cyanide from cassava wastewater. Along with thermostability, the immobilized enzyme showed 75% of cyanide biodegradation efficiency [146].

17α-ethinylestradiol is a synthetic hormone used in oral contraceptives, but it reaches in the aquatic system due to anthropogenic activities and through pharmaceutical effluents, hence causing great environmental impact. Previously, the laccase was immobilized on the copper and calcium chitosan–alginate composite for degradation of 17α-ethinylestradiol. The degradation of 17α-ethinylestradiol was more effective in a wider temperature and pH range as compared to free laccase [147]. Moreover, Bisphenol A is an endocrine-disrupting compound and is found in the sewage sludge, surface water, and industrial effluents. Previously, laccase absorbed on the anionically activated agarose was applied for the elimination of bisphenol A. The immobilized laccase showed more degradation of bisphenol A (BPA) at 100 mg/l concentration than of the free form of the enzyme and of initial able to degrade >90% BPA after 15 cycles [148]. From these results, it can be concluded that polymer immobilized laccase is a promising candidate for the degradation of BPA in terms of efficiency and reusability.

**Figure 8.**
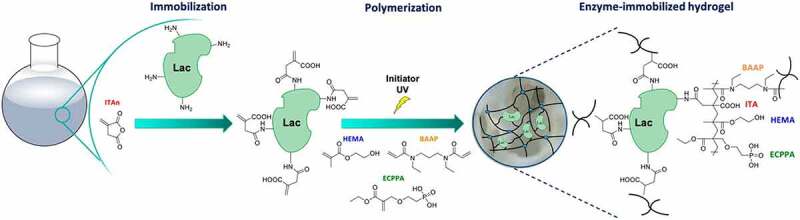
Schematic illustration of hydrogel preparation containing itaconic acid immobilized laccase. Reproduced with permission from Horn et al. 2021., applied polymer materials, 3, 2823–2834 [151].

In another study, crude laccase was entrapped inside the calcium alginate beads for the degradation of BPA. They reported that immobilized laccase retained up to 70% of removal efficiency up to 10 cycles of reuse, and after that starts it starts declining [149]. The decrease in removal efficiency by immobilized laccase was due to leakage of an enzyme from the alginate matrix. In a previous study, Bilal et al. [150] immobilized laccase on chitosan beads for the degradation of BPA, and it showed complete degradation of BPA (approximately 99%) after 150 min. This enzymatic bioremediation system could be helpful in the degradation of dyes present in the wastewater effluents. Previously, Horn and his coworkers immobilized a lacasse on the hydrogel containing itaconic acid for the reduction of trace compounds (*e.g*., BPA, triclosan, *p*-chlorophenol, paracetamol, diclofenac) in wastewater. The immobilized enzyme showed a maximum reduction of triclosan (>90%) (Figure 8) [151]. Furthermore, a proteolytic enzyme known as papin having the ability to bind with metal ions due to the presence of sulfhydryl group in the active center was immobilized in alginate beads for removal of lead. The immobilized enzyme was found to remove 98.88% of lead at 10 mg/mL of total concentration [152]. Indeed, Qu and his worker entrapped a microbial lipase onto chitosan nanoparticles for removing nickel impurities from waste water. The immobilized enzyme was re-used up to 20 cycles for the removal of nickel [153]. Based on these results, the polymer immobilized enzyme can be highly recommended for bioremediation of pollutants released from industrial wastewater, due to substantial improvement in water quality after treatment with an immobilized enzyme. Future research should focus on developing a novel biocatalytic system for bioremediation of large-scale textile wastewater in a sustainable way.

#### 5.3.2. Greenhouse gas capture using polymeric matrics immobilized enzyme

**Figure 9.**
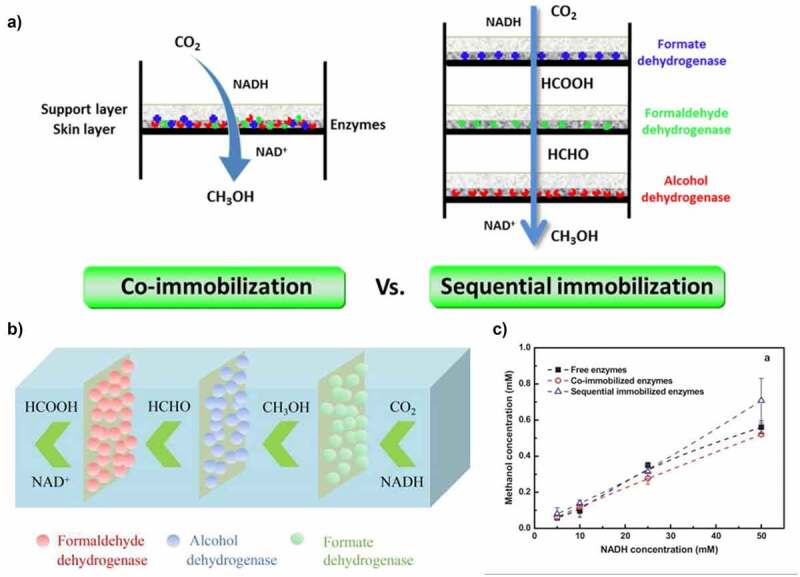
A) Conversion of CO_2_ to methanol using co-immobilization and sequential immobilization methods. B) The schematic representation of the sequential immobilization system. C) production of methanol using a free and immobilized enzyme. Reproduced with permission from Luo et al. 2015., New Biotechnology, 32, 319–327; .[163] and Cen et al. 2019., Advance synthesis and Catalysis, 361, 5500–5515 [164].

The constant rise in greenhouse gases (GHGs) is causing serious threats to living beings and the environment. CO_2_ is one of the main contributors to greenhouse effect and the emissions of CO_2_ from electric power generation, coal-burning stations, and automobiles increase the atmospheric CO_2_ concentration, thus impart in global warming [154–156]. In this context, CO_2_ conversion into value-added products, such as methanol, calcium carbonates, formic acid using microbial enzymes could be effective to meet the energy demand of industries [14,157,158]. Notably, in 2020, the atmospheric CO_2_ concentration was found to be 417 ppm, which critically affects human beings’ health [141,159–161]. Enzymes, such as carbonic anhydrase (CA), alcohol dehydrogenase (ADH), formaldehyde dehydrogenase (FaldDH), and formate dehydrogenase (FDH) have been utilized for the conversion of CO_2_ into value-added products [3,162]. The polymeric matrices should have a good affinity for substrate and mass transfer of CO_2_ for enhancing its capture. For example, ADH, FaldDH, FDH were immobilized in the polymeric membrane using co-immobilization and sequential immobilization method for the production of methanol from CO_2_. The sequential immobilization method displayed higher methanol production, as this method allows the operational conditions to be optimized at each step and overcome the diffusion resistance among enzymes (Figure 9) [163,164]. Furthermore, Sharma et al. [165] immobilized CA of *P. fragi* on chitosan for biomimetic CO_2_ sequestration. Notably, a more than two-folds increase in the formation of calcium carbonate was observed with the immobilized enzyme. The calcium carbonate formed during CO_2_ conversion can be used as raw material for cement, iron, ceramics, steel, and glass production units.

Furthermore, Xu and his coworkers [166] encapsulated alcohol dehydrogenase, formate dehydrogenase, and formaldehyde dehydrogenase in silica alginate hybrid gel for conversion of CO_2_ to methanol. The resultant biocatalyst exhibited enhanced methanol yield that might be due to the formation of a suitable immobilization microenvironment *i.e*., high hydrophilicity, ideal diffusion, and moderate flexibility required for a substrate. Methanol, exploited as an energy source for manufacturing daily need products, can be used in electricity generation and important raw material for industrial applications [167]. A novel method for encapsulation of formate dehydrogenase in alginate-silica gel for conversion of CO_2_ into formic acid was reported. The resultant biocatalyst exhibited a high yield of formic acid *i.e*., 95.6%, which indicates that biocatalyst retained the conformational flexibility after immobilization [168]. Recently, Pietricola and his coworkers immobilized *Candida boidinii* formate dehydrogenase onto mesoporous silica and catalyzed the reduction of CO_2_ into formic acid. The immobilized enzyme exhibited higher immobilization yield, thermal stability but the low yield of formic acid was obtained. As the enzymatic reduction reaction of CO_2_ is reversible, so formed formic acid is converted back into CO_2_ which results in a low yield of formic acid [169]. To upscale the CO_2_ conversion experiment various challenges need to be overcome such as various impurities present in flue gas including nitrous oxide and sulfur trioxide that may affect the enzyme performance [170,171]. Furthermore, it is necessary to study the complex interaction between polymeric matrices and enzymes especially stabilization of enzyme confirmation while designing the immobilization system for CA. We expect that in the future, researchers might attain sustainable synthesis of value-added products using polymer immobilized enzymatic systems.

## 6. Challenges and future directions

Moreover, several polymeric matrices and their composites with noteworthy properties and unique structures have been designed for enzyme immobilization and the utilization of suchadvanced materials could potentially enhance the biocatalytic properties. Despite of the huge success of immobilized enzymes, the main challenges that needed to be overcome:

- In up-scaling bioprocess using immobilized enzyme is mostly cost-driven, so before immobilization, the economic evaluation of all, such as enzyme, reactors, downstream processing, and matrices for immobilization, should be carefully investigated [115].
- The effective approaches for the development of enzyme immobilization-stabilization and availability of sustainable industrially applicable enzymes are a necessity of green enzyme processes.
- The environmental impact of the immobilization process should be low, as crosslinking chemicals are often used in the immobilization process.
- To expand the usage of polymeric matrices for the immobilization of enzymes, it is important to obtain a deeper insight into the effects of polymeric matrices on the function, structure and kinetics of an enzyme. This detailed study may prove to be helpful to develop a stable biocatalytic system.
- To attain such goals, future research should focus on developing new generations of immobilized biocatalysts by taking the advantage of genetic manipulations, bioinformatics, organic chemistry, and computational chemistry. Most importantly, the incorporation of these techniques could help in better visualization of conformational changes that happen in immobilized enzymes, matrices, and enzyme binding sites. Before empirical techniques, applying molecular stimulations techniques can minimize costly trials, investigation error, and time consumption for the development of versatile immobilized enzymes. Hence, it will be simpler to develop a well functional immobilized enzyme that can meet future needs.

## 7. Conclusion

The comprehensive review examined that the enzyme immobilization on polymeric matrices is a powerful way to create a novel biocatalytic system with various industrial applications. The utilization of biopolymers as matrices for enzyme immobilization from the utmost plentiful and sustainable resources also addresses the rising global requirements. Polymers and their composite are emerging as host materials for the immobilization of enzymes because of their numerous features, such as nontoxicity, unique structure, and stability. However, the use of only polymeric matrices cannot satisfy all the properties required for enzyme immobilization, and so surface functionalization represents a successful approach to introduce ideal properties that are different from the native matrix. The polymeric matrices are often preferred for enzyme immobilization due to the high possibility of surface modification and availability. Additionally, enzyme immobilization on to functionalized polymers enhances the robustness and durability of the biocatalyst for its reuse. The modified polymers should be studied more to find better matrix for enzyme immobilization. We believe in the future, the polymer immobilized enzyme will be more reusable, affordable and reliable, and widely available in industries and laboratory.
